# Mild/moderate phenotypes in AADC deficiency: Focus on the aromatic amino acid decarboxylase protein

**DOI:** 10.1002/jimd.12791

**Published:** 2024-08-21

**Authors:** Giovanni Bisello, Rossella Franchini, Cristian Andres Carmona Carmona, Mariarita Bertoldi

**Affiliations:** ^1^ Department of Neuroscience, Biomedicine and Movement Sciences University of Verona Verona Italy

**Keywords:** AADC deficiency, aromatic amino acid decarboxylase, compound heterozygosis, genotype–phenotype correlation, mild/moderate phenotype

## Abstract

AADC deficiency is a severe neurometabolic inherited rare disorder due to the absence or decrease of dopamine and serotonin levels, causing deep motor and neurodevelopmental impairments. The disease is often fatal in the first decade of life, and pharmacological treatments (dopamine agonists, pyridoxine, and monoamine oxidase inhibitors as the first‐line choices) can only alleviate the symptoms. Gene therapy surgery is now available for severe patients in the European Union and the United Kingdom, and follow‐up data witness encouraging improvements. In the past few years, mostly due to the increased awareness and knowledge of AADC deficiency, together with newborn screening programs and advancements in methods for genetic diagnosis, the number of mild/moderate phenotypes of AADC deficiency patients has increased to 12% of the total. A review of the genotypes (homozygous/compound heterozygous) of AADC deficiency mild/moderate patients is presented here. The pathogenicity classification of each genetic variant is discussed. Then, we focused on the type of AADC protein possessed by patients and on the predictable structural score of the homodimeric/heterodimeric species of each protein variant. Since the terminology used for genetic and protein variants is the same, we highlighted how it could be misleading. We analyzed the loss‐of‐function as a fold‐change decrease of activity of the recombinant purified AADC enzyme(s) theoretically synthesized by mild/moderate patients. A minimal residual *k*_*cat*_ of 8% and/or *k*_*cat*_/K_m_ of 1% seems necessary to avoid a severe disease manifestation. Overall, this cluster of mild/moderate patients needs consideration for a more appropriate and aimed therapeutic approach.

## 1 INTRODUCTION

Monogenic rare inherited neurometabolic disorders are due to modifications in a single gene coding for a protein/enzyme involved in the metabolism of essential neuromodulators. Given the individual genotypes typical of inherited monogenic diseases and the related phenotypic heterogeneity, such disorders present broad clinical presentations. The clinical phenotype cannot entirely depend on the modified protein, whose molecular characterization could, however, contribute to the understanding of genotype–phenotype correlation. Phenylketonuria is paradigmatic in this sense: mutations on the phenylalanine hydroxylase gene well describe the biochemical phenotype.^1^, ^2^ At the same time, intellectual disability can be influenced by multiple factors directly or indirectly dependent on the modified enzyme.^1^, ^2^ Aromatic amino acid decarboxylase (AADC) deficiency (MIM #608643) is a monogenic neurodevelopmental disease due to mutations occurring in the *DDC* gene (MIM #107930) coding for the AADC enzyme. AADC synthesizes the monoamine neurotransmitters dopamine and serotonin from L‐dopa and L‐5‐hydroxytryptophan, respectively.^3^ The lack of these essential neurotransmitters significantly impacts motor and developmental activities. The presentation of the disorder is often severe,^4^ consisting of deep motor and neurodevelopmental impairments coupled with other autonomic symptoms.

The common hallmark is represented by the oculogyric crises, triggering severe pain and stress in patients. The pharmacological support aims to alleviate symptoms and consists of: (i) pyridoxine supplementation since AADC depends on the derivative pyridoxal 5′‐phosphate (PLP) for activity, (ii) dopamine agonists and (iii) monoamine oxidase inhibitors.^4^, ^5^ Response to these treatments is feeble, especially in severe cases (the majority). A gene therapy addressed to intraputaminal delivery of eladocagene exuparvovec (Upstaza®) (the human adeno‐associated virus (AAV)‐cDNA for wild‐type (WT) enzyme) has been approved in the European Union (in 2022) and in the United Kingdom (in 2023) targeted to patients older than 18 months with a severe clinical phenotype (no sit, no walk, and no head control) and a confirmed metabolic, molecular and genetic diagnosis of AADC deficiency.^5^

## 2 MILD/MODERATE AADC DEFICIENCY PHENOTYPES AND THE GENETIC DETERMINATION OF VARIANT PATHOGENICITY

In addition to the predominant severe clinical phenotype, few patients present with a mild/moderate form of the disorder. The definition of mild and moderate has been provided by the guidelines^4^ and has been recently further specified.^6^ In particular, a mild phenotype by the guidelines^4^ refers to a mild presentation of intellectual, developmental, and motor capabilities. In contrast, a severe phenotype is characterized by the inability to stand and to sit without assistance, by the lack of head control, in addition to neurodevelopmental symptoms. A moderate phenotype presents with symptoms that lie in severity between mild and severe phenotype. Recently, Pearson et al.^6^ specified further by defining a patient able to walk independently as mild, a patient that does not reach minimal attainments of developmental milestones as severe, and intermediate cases as moderate.

While up to 2019, the number of diagnosed mild/moderate patients was 21, in recent years (from 2020 to March 2024), the number doubled with 22 newly published mild/moderate cases (references to mild/moderate patients listed by year of publication are reported in Tables 1 and 2). This is probably due to several causes. First, the increase in awareness and knowledge of this disease; second, the acceleration in genetic diagnosis by next‐generation sequencing techniques, including whole exome and genome sequencing, applied to cohorts of patients with undiagnosed or heterogeneous neurodevelopmental conditions.^18^ Finally, newborn screening programs, based on the detection of the 3‐O‐methyldopa (3OMD) marker in dried blood spots,^32^, ^33^, ^34^, ^35^, ^36^ increased the number of new AADC deficiency patients.

**TABLE 1:** Homozygous genotypes, phenotypes, and AADC protein species of mild/moderate AADC deficiency patients. Each line refers to identified individual patients. Transcript ID is NM_001082971.2 (ClinVar); protein ID is P20711 (UniProt).

Year	Phenotype	Genotype (cDNA)	ACMG score^7^	AADC homodimers	3D score^8^
2004	Mild	c.[749C > T]; [749C > T]^9^	P/P	p.S250F/p.S250F	LP
	Mild	c.[749C > T]; [749C > T]^9^	P/P	p.S250F/p.S250F	LP
	Mild	c.[304G > A];[304G > A]^10^, ^11^	P/P	p.G102S/p.G102S	P
	Mild	c.[304G > A];[304G > A]^10^, ^11^	P/P	p.G102S/p.G102S	P
	Mild	c.[304G > A];[304G > A]^10^, ^11^	P/P	p.G102S/p.G102S	P
2009	Mild	c.[206C > T];[206C > T^11^, ^12^	P/P	p.T69M/p.T69M	P
2014	Mild	c.[665 T > C];[665 T > C]^13^	P/P	p.L222P/p.L222P	P
2015	Mild	c.[1357C > T];[1357C > T]^14^, ^15^	n.d./n.d.	p.R453C/p.R453C	P
	Mild	c.[1357C > T];[1357C > T]^14^, ^15^	n.d./n.d.	p.R453C/p.R453C	P
	Mild	c.[1357C > T];[1357C > T]^14^, ^15^	n.d./n.d.	p.R453C/p.R453C	P
2020	Moderate	c.[201 + 5G > C];[201 + 5G > C]^6^, ^7^, ^a^	LP/LP	?	n.d.
2021	Moderate mild	c.[304G > A];[304G > A]^16^ c.[44A > G]; c.[44A > G]^17^	P/P LP/LP	p.G102S/p.G102S p.D15G/p.D15G	P LP^7^
2023	Mild	c.[1385G > A];[1385G > A]^18^, ^19^	n.d./n.d.	p.R462Q/p.R462Q	LB^b^
2024	Mild	c.[941 T > C];[941 T > C]^20^	n.d/n.d.	p.M314T/p.M314T	n.d.
		Total number of patients = 15			
		Total number of genotypes = 9			

Abbreviations: LB, likely benign; LP, likely pathogenic; n.d., not determined; P, pathogenic.

^a^ Denotes an intronic variant with possible splicing site alteration or no effect.

^b^ This work.

**TABLE 2:** Compound heterozygous genotypes, phenotypes, and AADC protein species of patients with mild/moderate AADC deficiency.

Functionally hemizygotes					
Year	Phenotype	Genotype (cDNA)	ACMG score^7^	AADC homodimers	3D score^8^
2007	Mild	c.[714 + 4A > T]; [853C > T]^21^	P/P	p.R285W/p.R285W	P
	Mild	c.[714 + 4A > T]; [853C > T]^21^	P/P	p.R285W/p.R285W	P
2010	Mild	c.[19C > T]; [1222C > A]^22^	P/P	p.L408I/p.L408I	P
2014	Mild	c.[289delC]; [629C > T]^13^, ^15^	VUS/VUS	p.P210L/p.P210L	LP
2015	Mild	c.[105delC]; [710 T > C]^15^, ^23^	P/LP	p.F237S/p.F237S	P
	Mild	c.[105delC]; [710 T > C]^15^, ^23^	P/LP	p.F237S/p.F237S	P
2016	Mild	c.[714 + 4A > T]; [752 T > C]^15^, ^24^	P/LP	p.F251S/p.F251S	P
2020	Moderate	c.[714 + 4A > T]; [179 T > C]^6^, ^15^	P/P	p.V60A/p.V60A	P
	Moderate	c.[476C > T];?^6^	LP/LP	p.A159V/p.A159V	LP
	Moderate	c.[1021 + 1G > A]; [299G > C]^16^, ^25^	LP/LP	p.C100S/p.C100S	P
2021	Mild	c.[19C > T]; [299G > C]^26^	LP/LP	p.C100S/p.C100S	P
		Total number of patients = 11			
		Total number of genotypes = 8			

		Compound heterozygotes		
Year	Phenotype	Genotype (cDNA)	ACMG score^7^	AADC species
2012	Moderate	c.[478C > T];[1040G > A]^27^	LP/P	p.R160W/p.R160W p.R347Q/p.R347Q p.R160W/p.R347Q
2013	Mild	c.[97G > C];[1385G > C]^28^	LP/LP	p.V33L/p.V33L p.R462P/p.R462P p.V33L/p.R462P
2014	Mild	c.[260C > T];[446G > A]^13^	P/LP	p.P87L/p.P87L p.S149T/p.S149T p.P87L/p.S149T
2019	Mild	c.[272C > T];[1228 T > C]^29^	LP/LP	p.A91V/p.A91V p.C410G/p.C410G p.A91V/p.C410G
2020	Mild	c.[367G > A];[876G > A]^6^, * ^a^	LP/VUS	p.G123R/p.G123R p.E292E/p.E292E p.G123R/p.E292E
	Mild	c.[367G > A];[876G > A]^6^, * ^a^	LP/VUS	p.G123R/p.G123R p.E292E/p.E292E p.G123R/p.E292E
	Mild	c.[206C > T];[1337 T > C]^6^	P/LP	p.T69M/p.T69M p.L446P/p.L446P p.T69M/p.L446P
	Mild	c.[367G > A];[734C > T]^6^	LP/LP	p.G123R/ p.G123R p.T245I/ p.T245I p.G123R/p.T245I
	Mild	c.[843C > G];[1085 T > C]^6^, ^12^	LP/LP	p.C281W/p.C281W p.M362T/p.M362T p.C281W/p.M362T
	Mild	c.[260C > T];[446G > C]^6^	P/LP	p.P87L/p.P87L p.S149T/p.S149T p.P87L/p.S149T
	Moderate	c.[260C > T];[446G > C]^6^	P/LP	p.P87L/p.P87L p.S149T/p.S149T p.P87L/p.S149T
	Moderate	c.[478C > G];[565G > T]^16^, ^25^	LP/LP	p.R160G/p.R160G p.D189Y/p.D189Y p.R160G/p.D189Y
	Moderate	c.[2 T > C];[277A > G]^16^, * ^b^	LP/LP	p.M1K/p.M1K p.M93V/p.M93V p.M1K/p.M93V
2021	Mild	c.[202G > A];[254C > T]^17^	LP/LP	p.V68M/p.V68M p.S85L/p.S85L p.V68M/p.S85L
	Mild	c.[202G > A];[254C > T]^17^	LP/LP	p.V68M/p.V68M p.S85L/p.S85L p.V68M/p.S85L
2023	Mild	c.[1040G > A];[1172 T > C]^30^	P/VUS	p.R347Q/p.R347Q p.L391P/p.L391P p.R347Q/p.L391P
2024	Mild	c.[679G > C]; [1040G > A]^31^	VUS/P	p.E227Q/p.E227Q p.R347Q/p.R347Q p.E227Q/p.R347Q
		Total number of patients = 17		
		Total number of genotypes = 13		

*Note*: Each line refers to identified individual patients. Functionally hemizygotes are characterized by an allele that does not synthesize a complete AADC polypeptide chain due to alterations in splicing, insertions, or deletions that determine a premature stop codon and an incomplete, not mature chain. Transcript ID is NM_001082971.2 (ClinVar); protein ID is P20711 (UniProt).

Abbreviations: LP, likely pathogenic; n.d., not determined; P, pathogenic; VUS, variant of unknown significance.

* Genotypes that could give rise to functionally hemizygotes since there is an intronic variant^a^ or the first codon alteration^b^. Since they present symptoms, they cannot be considered healthy heterozygous carriers.

The pharmacological approach for mild/moderate patients is the same as for severe ones, except for gene therapy, which is currently not contemplated. A recent review discussed gene augmentation therapy in mild AADC deficiency patients since, from this treatment, it is conceivable that they could reach a nearly normal life.^37^

Tables 1 and 2 list the number of diagnosed mild/moderate patients and their identified genotypes. Even if it is difficult to accurately count the number of patients since many of them may appear in different papers, we tried to filter them and have a reliable estimate of the number of mild/moderate patients to the total ones (43/348^7^) with a measured value of 12.3%.

The mild appearance of the disease is associated more frequently with a compound heterozygous genotype (21/30; 70%) and is not related to the type of DNA mutation and amino acid protein substitution. Among the mild patients, the very severe homozygous genotype of Taiwanese and South‐Asian inheritance (c.[714 + 4A > T]; c.[714 + 4A > T]), which represents about 21% of all AADC deficiency cases,^7^ is absent. This is expected since this splicing mutation is foreseen to synthesize, if any, a 238 amino acid long‐truncated AADC polypeptide. Nevertheless, the c.[714 + 4A > T] genetic variant has been identified in four compound heterozygotes that, on the other allele, carry a point mutation.^6^, ^21^, ^24^ Similar to genotypes associated with severe patients, most variants leading to milder phenotypes are point mutations, causing single amino acid substitutions.

Notably, individual variants of Mendelian disorders have been classified by the American College of Medical Genetics and Genomics (ACMG) with precise terminology following defined discriminating criteria based on population data, computational data, functional data, and segregation data,^38^ as pathogenic (P), likely pathogenic (LP), likely benign (LB), benign (B), or variant of unknown significance (VUS).^38^ In AADC deficiency, most genetic variants of the identified genotypes have been described as P or LP.^7^ Restricting the examination to genotypes and variants of mild/moderate patients, P and LP variants represent a high percentage unless a few VUS (Tables 1 and 2).

Remarkably, according to the ACMG recommendations, the classification is not necessarily related to the severe or mild/moderate metabolic (and clinical) phenotype since this is primarily due to the activity of the AADC enzyme, which is derived from the corresponding gene variant.^8^

## 3 VARIANT PATHOGENICITY FROM A PROTEIN PERSPECTIVE

**FIGURE 1:**
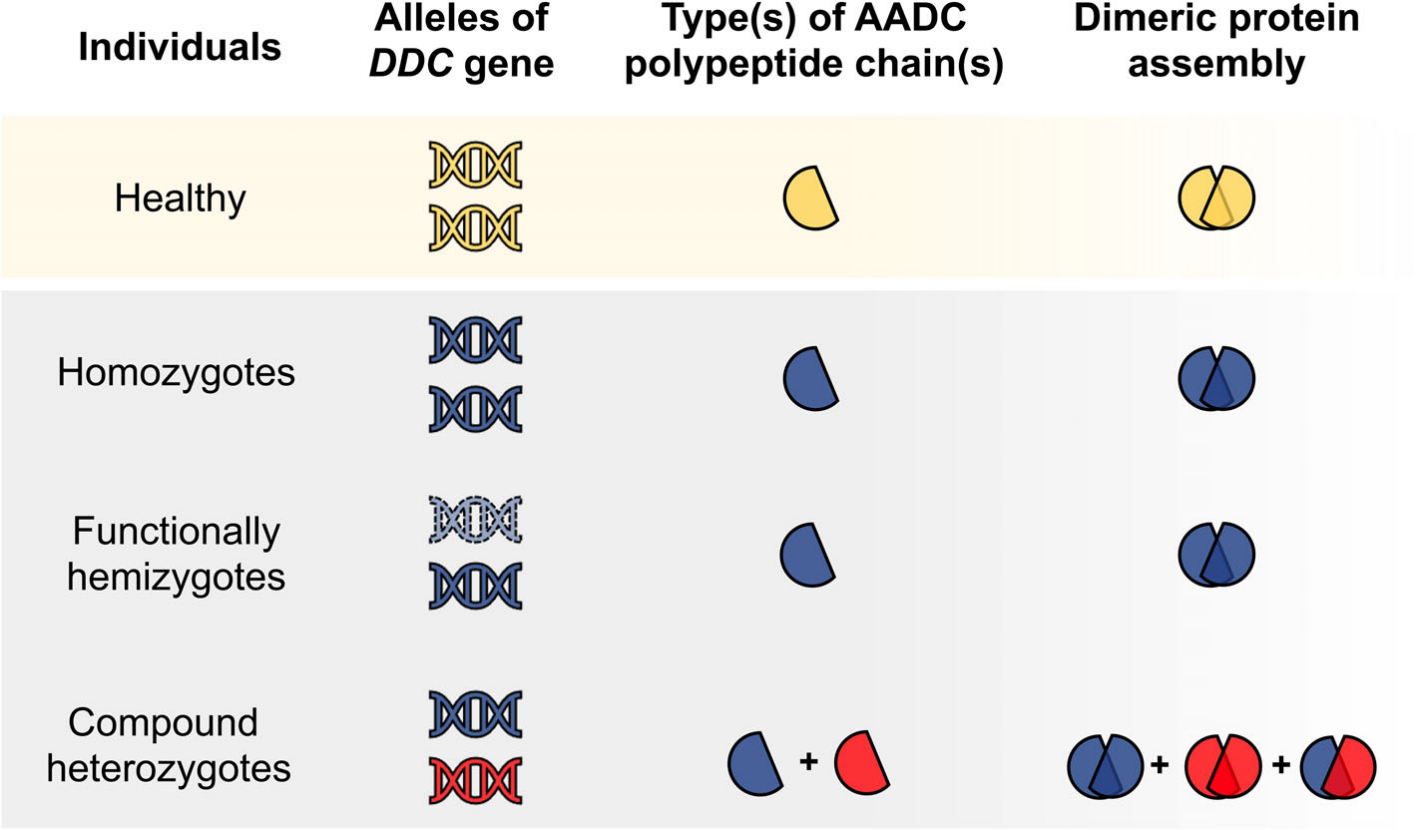
Possible combinations of AADC polypeptide chains in homozygous, functionally hemizygous and compound heterozygous AADC deficiency patients. Healthy individuals with no mutated *DDC* alleles and wild‐type AADC protein are represented in yellow. In homozygotes, the same mutation occurs on both alleles, determining the production of the same type of AADC polypeptide chain and only one type of homodimeric AADC variant (blue). Functionally hemizygotes possess an allele (dashed blue) that does not produce a complete AADC polypeptide chain (not present as synthesized species) and a second allele carrying one mutation, giving rise to only one type of AADC dimeric variant (blue). The two alleles of compound heterozygous patients present different mutations (represented as blue and red). They can synthesize two different types of AADC polypeptide chain, giving rise to three different combinations of such chains in the dimeric assembly of the AADC dimer: Two different homodimers (totally blue or red) and one heterodimer (blue‐red).

A structural alteration of an enzyme variant could lead to a functional consequence that increases or decreases the efficiency of catalysis. A prediction of “pathogenicity” based on a three‐dimensional (3D) score for each AADC protein variant has been recently developed to predict the impact on the structure that is necessarily related to the function. This 3D score is based on several criteria: evolutionary conservation of the individual modification, predictable structural impact in the dimeric AADC species, alteration in polarity and molecular volume of the substituted amino acid, and chemical nature of the substitution.^7^, ^8^ The relative weight of the combination of these features gives P, LP, LB, or B outcomes.^7^, ^8^ Notably, the 3D protein score was expressed using the same terminology recommended by the ACMG criteria,^7^, ^8^ but the significance was dissimilar. The 3D score, different from the genetic variants, is based on the predicted possible impact on the AADC obligate functional dimer. It follows that genotype dictates the type(s) of AADC polypeptide chains that can associate to form the active dimeric species. Homozygous genotypes can give rise to one AADC homodimeric protein variant,^39^ which represents the only AADC species in such patients (Figure 1). The eight homozygous genotypes of Table 1 produce AADC homodimers whose structural 3D scores are mostly P/LP. As for compound heterozygous genotypes (Table 2), a first distribution could be made between those giving rise to only one AADC protein species (the “functionally hemizygotes”) and those theoretically synthesizing a battery of AADC species: two homodimer and one heterodimer variants (Figure 1). The functionally hemizygotes can be considered as the homozygous ones from a protein point of view since each genotype can lead to only one AADC protein variant. The 3D scores of their AADC proteins are mainly P/LP (Table 2), as those synthesized from the homozygotes.

The functionality of the AADC protein pool possessed by compound heterozygous patients (with one missense mutation on each allele) is likely due to the added contribution of each species: homodimers and heterodimer. Moreover, the two variants can positively or negatively complement.^39^ To give a value of predicted pathogenicity to the heterodimer, a 3D score for all AADC enzyme species present in mild/moderate compound heterozygotes has been elaborated (Table 3). The prediction for the heterodimeric species is based on the relative position of the amino acid substitutions in the dimeric structure and the number of affected active sites in this species.^7^ Interestingly, P/LP 3D protein scores also predominate in the compound heterozygous genotypes of mild/moderate patients.

**TABLE 3:** Prediction of the structural effects of AADC protein variants corresponding to the genotypes of compound heterozygous patients^**a**^.

	3D protein score^7^, ^8^, ^b^		number of affected active sites^c^			3D heterodimer score^e^
Compound heterozygotes	Homo dimer 1	Homo dimer 2	Homo dimer 1	Homo dimer2	Heterodimer^d^	
p.R160W/p.R347Q	P	P	0	2	1	LP
p.V33L/p.R462P	LP	P	0	0	0	LB
p.P87L/p.S149T	LP	LP	2	2	1	LP
p.A91V/p.C410G	P	P	2	0	1	LP
p.G123R/p.E292E	P	?	2	0	1	LP
p.T69M/p.L446P	P	LP	0	0	0	LB
p.G123R/p.T245I	P	P	0	2	1	LP
p.C281W/p.M362T	P	P	0	2	1	LP
p.R160G/p.D189Y	P	LP	0	0	0	LB
p.M1K/p.M93V	?	LP	0	0	0	LB
p.V68M/p.S85L	LP	LP	0	2	1	LP
p.R347Q/p.L391P	P	LP	2	0	1	LP
p.E227Q/p.R347Q	LB	P	0	2	1	LP

*Note*: ? is related to the unknown product of synonymous or first codon alteration.

^a^ All data are from^7^, ^8^ except for p.G123R/p.E292E, p.M1K/p.M93V, p.R347Q/p.L391P and p.E227Q/p.R347Q.

^b^ For the homodimers, pathogenic (P), likely‐pathogenic (LP), and likely‐benign (LB) predictions depend on a combinatory evaluation of evolutionary conservation and structural alteration in polarity and molecular size of the substituted amino acid, as reported.^7^

^c^ In the homodimers: 0 means that the identical amino acid substitutions in both monomers of the homodimer are far from the active site region; 2 means that both active sites of the homodimer are affected by the identical amino acid substitutions on the two different monomers.

^d^ In the heterodimer: 0 means that the amino acid substitutions in both monomers of the heterodimer are far from the active site region; 1 means that one active site of the heterodimer is affected by the combination of amino acid substitutions on the two different monomers, whereas the other active site is not affected; 2 means that both active sites of the heterodimer are affected by the combination of amino acid substitutions on the two different monomers.

^e^ The numerical score 0 was converted into LB, the score 1 into LP and the score 2 into P according to.^7^

Overall, genetic classifications (by ACMG criteria) and structural predictions (by 3D protein scores) of variants do not allow us to distinguish severe from mild/moderate phenotypes. In addition, using the same terminology to define the pathogenicity of genetic and protein variants can lead to a misunderstanding. From a biochemical point of view, an enzymatic variant is better defined based on its functionality, i.e., residual activity.

## 4 LOSS‐OF‐FUNCTION OF AADC PROTEIN VARIANTS ASSOCIATED WITH MILD/MODERATE PHENOTYPES

**FIGURE 2:**
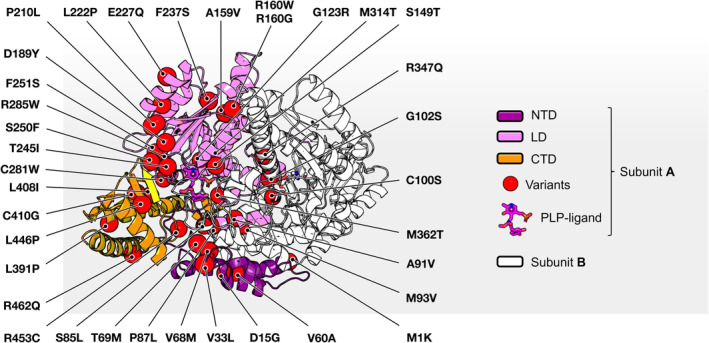
In silico visualization of the position in the crystal structure of AADC variants of mild/moderate AADC deficiency patients. Ribbon representation of the human holoAADC (pdb ID 8ORA).^40^ Subunit A is colored by domains: N‐terminal domain (NTD, amino acids 1–85), large domain (LD, amino acids 86–360), and C‐terminal domain (CTD, amino acids 361–480) are colored purple, pink, and orange respectively. Subunit B is white. The PLP‐Dopa analog is represented as sticks with CPK color code. The position of the amino acid variants in mild/moderate patients of AADC deficiency are labeled and represented as red spheres. The image was rendered by PyMol software (Schrödinger).

To refine the knowledge of the structural effect predicted for each protein variant, we mapped the position of each substituted amino acid of AADC variants synthesized by mild/moderate patients on the AADC dimer (Figure 2). Notably, AADC comprises two identical subunits assembled in a geometrical antisymmetric manner with swapped stretches in the functional oligomer.^40^ It has been recently reported that the deepest loss‐of‐function is related to variants affecting the active site/loops in contact with it and regions far from the active site but responsible for the enzyme structural dynamics.^40^ AADC variants of mild/moderate patients mainly belong to protein regions not involved in dramatic loss‐of‐function. In more detail, a high percentage (56%) of the 36 AADC protein variants present in mild/moderate patients belongs to the large domain and not to the active site or regions involved in structural dynamics control, essential for catalysis.^40^

As a further step, we associated the published functional data regarding the kinetic parameters of AADC variants of mild/moderate AADC deficiency patients to the respective genotypes (Tables 1 and 2). It should be underlined that patients with the same genotype theoretically give rise to the same type(s) of AADC enzyme variants. Notably, a discrete number of AADC variants have been obtained as purified recombinant species and functionally characterized. Their relative decreases in the catalytic constant (*k*_*cat*_) and the increase in the Michaelis–Menten constant (K_m_) are compared with the wild‐type (WT) human AADC (Table 4). The *k*_*cat*_ is related to the capability of the enzymatic variant to carry out catalysis at saturating L‐Dopa concentration, under experimental conditions similar to those used to evaluate AADC activity of AADC deficiency patients in plasma.^42^ Thus, the determined values of *k*_*cat*_ reflect the residual activity of the AADC variants.

**TABLE 4:** Fold change of *k*_*cat*_ and *k*_*cat*_/K_m_ decrease and K_m_ increase of AADC deficiency variants present in homozygous, functionally hemizygous and compound heterozygous patients.

Dimeric AADC variants	Fold‐change decrease in *k*_*cat*_ ^40^	Fold‐change increase in K_m_ ^40^	Fold‐change decrease in *k*_*cat*_/K_m_ ^40^
Homozygous^a^			
p.D15G/p.D15G	n.d.	n.d.	n.d.
p.T69M/p.T69M	2.2	4.2	9.1
p.G102S/p.G102S	6.3	10.9	69.1
p.L222P/p.L222P	n.d.^15^	n.d.^15^	n.d.^15^
p.S250F/p.S250F	3.6	2	7.2
p.M314T/p.M314T	n.d.	n.d.	n.d.
p.R453C/p.R453C	11.3	3.6	41.3
p.R462Q/p.R462Q	n.d.	n.d.	n.d.
Functionally hemizygous^a^			
p.V60A/p.V60A	8.2	1.5	11.9
p.C100S/p.C100S	1.1	3.8	4.2
p.A159V/p.A159V	n.d.	n.d.	n.d.
p.P210L/p.P210L	1.7	1.1	1.9
p.F237S/p.F237S	n.d.^15^	n.d.^15^	n.d.^15^
p.F251S/p.F251S	1.9	0.8	1.5
p.R285W/p.R285W	3.7	1.5	5.3
p.L408I/p.L408I	9.7	16.2	157.7
Compound heterozygous^a^, ^b^			
p.R160W/p.R160W	6.8	17.7	120.3
p.R347Q/p.R347Q	87.4	4.5	389.2
p.R160W/p.R347Q	n.d.^41^	n.d.^41^	n.d.^41^
p.C281W/p.C281W	n.d.^12^	n.d.^12^	n.d.^12^
p.M362T/p.M362T	1.7	1.6	2.7
p.C281W/p.M362T	0.4	1.1	3.2
p.A91V/p.A91V	915.7	1.4	1248.8
p.C410G/p.C410G	1.7	2.5	4.2
p.A91V/p.C410G	20	n.d.	n.d.
p.E227Q/p.E227Q	1.1^31^	1.1^31^	1.2^31^
p.R347Q/p.R347Q	528.6^31^	4.5^31^	389.2^31^
p.E227Q/p.R347Q	1.6^31^	0.9^31^	1.3^31^

*Note*: Values are reported as fold‐change with respect to the WT species. Data are from,^40^ unless otherwise stated.

Abbreviation: n.d., not determined.

^a^ Homozygous and functionally hemizygous patients synthesize one dimeric AADC variant (one dimeric purified recombinant species), whereas compound heterozygous patients are theoretically able to synthesize three dimeric AADC species (three dimeric purified recombinant species).

^b^ Values are reported for the AADC protein population species for which at least 2 out of 3 *k*_*cat*_ and K_m_ values have been calculated.

Interestingly, data suggest that a threshold of activity seems necessary to ensure a mild/moderate phenotype. In homozygotes and functionally hemizygotes, a loss‐of‐function not higher than 12‐fold (about 8% of residual activity) is compatible with the presence of a mild/moderate phenotype. In compound heterozygotes, since the AADC protein population is made of three species, it is sufficient that at least one of them maintains an activity higher than about 15% (6.8‐fold decrease) to generate a mild/moderate phenotype.

In addition to the catalytic constant, information regarding L‐Dopa affinity could also be relevant from a pharmacological point of view. Indeed, guidelines^4^ suggest L‐Dopa administration as an optional treatment for patients that present amino acid substitutions in the substrate binding site that could alter L‐Dopa binding. A recent paper^26^ reports the amelioration induced by L‐Dopa treatment in an iPSC model deriving from a mild patient.

It has been recently demonstrated that variants belonging to the dimer interface at the interconnection of the two active sites of the dimer can profoundly affect L‐Dopa binding.^40^ We observed that L‐Dopa affinity values of AADC homodimeric and heterodimeric species present in mild/moderate patients decrease by no more than 20‐fold. Notably, the combined value (*k*_*cat*_/K_m_) reflects the so‐defined catalytic efficiency of each AADC variant. Although the *k*_*cat*_/K_m_ values of AADC homodimers present in homozygous and functionally hemizygous mild/moderate patients do not decrease more than about 100‐fold (~1% of residual catalytic efficiency), for compound heterozygotes, it is sufficient that at least one species maintains 1% of catalytic efficiency to allow a mild/moderate phenotype (Table 4). Overall, the determined thresholds of *k*_*cat*_ and *k*_*cat*_/K_m_ compatible for a mild/moderate phenotype are tentative and should be better refined by increasing the number of characterized AADC variants.

Of course, conclusions cannot be drawn since nothing is known about allele dominance that would lead to different amounts of AADC polypeptide chain synthesis or AADC dimer intrinsic stability. In addition, the characterization of the entire AADC protein population of compound heterozygous patients is limited.

Notably, heterozygote carriers have an average AADC activity of 35%–40% of normal,^4^, ^43^ a value in the range of the AADC activity of most recombinant variants present in mild/moderate patients. The actual AADC activity necessary to discriminate healthy carriers from affected individuals seems to reside in a narrow range, and more investigation is needed to support these observations. Intriguingly, a recent paper focused on an Italian regional court of patients (Sicily) with various neurological disorders without identified etiology reported a high frequency of carriers of mutations associated with AADC deficiency.^44^ Thus, the genetic trait could be more widespread than recognized and pushes the neonatal screening^34^ as urgent to allow prompt identification and appropriate treatment that has a deep impact, especially at an early age.^4^ In addition, decisions regarding gene therapy treatment should also be considered for mild patients who could benefit from a remarkable life improvement.

## AUTHOR CONTRIBUTIONS

Giovanni Bisello (GB): Data analysis and interpretation. Manuscript drafting. Rossella Franchini (RF): Data analysis and interpretation. Manuscript drafting. Cristian Andres Carmona Carmona (CACC): Data analysis and interpretation. Manuscript drafting. Mariarita Bertoldi (MB): Study conceptualization, data interpretation, manuscript writing.

## FUNDING INFORMATION

This work has been supported by PTC Therapeutics IIS Grant, by PRIN2022 code: 2022BTMTP8 (Ministry of University and Research (MUR), National Recovery and Resilience Plan (NRRP)), and by #NEXTGENERATIONEU (NGEU) project MNESYS (PE0000006) – A Multiscale integrated approach to the study of the nervous system in health and disease (DN. 1553 11.10.2022) (Ministry of University and Research (MUR), National Recovery and Resilience Plan (NRRP)) to MB.

## CONFLICT OF INTEREST STATEMENT

Giovanni Bisello, Rossella Franchini, and Cristian Andres Carmona Carmona declare no conflict of interest. Mariarita Bertoldi received a research grant and speaker honorarium from the Drug Company PTC Therapeutics.

## ETHICS STATEMENT

This article is a review of existing data in literature and does not contain any studies with human or animal subjects performed by the authors.

## INFORMED CONSENT

This article does not contain any studies with human or animal subjects performed by any authors.

## Data Availability

All data are available upon request to the corresponding author.
